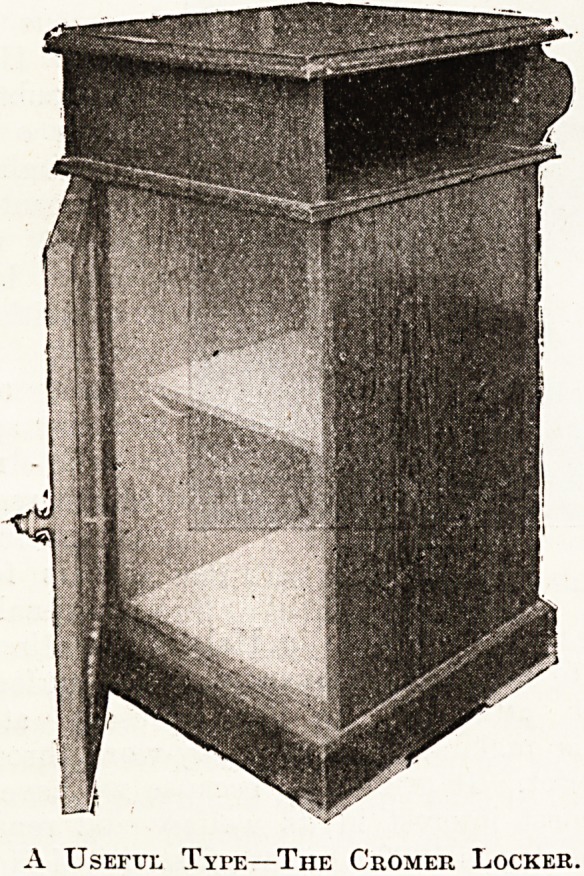# Reports on Hospitals of the United Kingdom

**Published:** 1913-11-29

**Authors:** Henry Burdett


### REPORTS ON
### Hospitals of the United Kingdom.

By SIR HENRY BURDETT, K.C.B., K.C.V.O.

SERIES III.

### HALSTEAD COTTAGE HOSPITAL.

The plan of this hospital, in spite of subsequent improvements, shows a lack of convenience owing to an original failure to make the best of the site. It still needs reconstruction to secure the maximum of facilities for the easy, economical, and efficient administration of every part of it. It contains at present insufficient accommodation for the nursing staff which it ought to have, having regard to the amount of valuable surgical work that is here carried on. The outlook from the wards is most attractive, and the equipment and furnishing of the building are reasonably good, with the exception of the mortuary, which is at present a place which cannot in any sense be regarded as a proper resting-place for the dead, or as one where the necessary pathological work can be undertaken or efficiently performed. In the course of these inspections we have often had to call attention to similar defects with regard to other mortuaries, and we hope that those at Halstead may speedily be remedied. The medical and hospital staff are to be congratulated upon the small mortality the returns exhibit, especially when the importance and character of the work are considered. The very fact that the deaths are so few should still more encourage someone with a high intelligence to make it his business to give this beautifully situated and admirably administered hospital a proper mortuary chamber and equipments.

The Cottage Hospital, Halstead.

### Finance.

It is a rule at this hospital that each patient should pay a minimum of 3s. per week during his treatment as an in-patient. The number of in-patients treated during the year 1912 was 103, and the patients' payments amounted to £38 2s. The average number of days' residence of each patient is not given at present in the report, so that we cannot work out the figures more in detail as we should like to do. We were informed that the Halstead people continuously complain of the hardship of in-patients contributing 3s. per week, but the Halstead people are in our experience nothing if not ungrateful, too critical of others, and too little conscious of their own limitations. They have reaped without appreciable acknowledgment priceless benefits through the in-patients' department as represented by its actual cost. All over the country except at Halstead, and we are familiar with the public opinion and the views of most if not all the districts which are blessed with an efficient hospital like that at Halstead, the inhabitants feel it a point of honour to exhibit gratitude to and appreciation of the men and women who, through the hospital, have ministered materially to the speedy recovery and return of the patients from sickness to health. It is surely time that a prosperous place like Halstead undoubtedly is should through its inhabitants exhibit repentance by the exhibition of more manliness and a better spirit of self-respect with regard to these matters. It is for the spiritual and other leaders of opinion in Halstead to stir up the people to a full consciousness of the unfortunate discredit attaching to their present attitude, which is making Halstead a by-word throughout the district. It grieves us because we have taken the deepest interest in its welfare and reputation since the day in 1883 when we first visited Halstead to take our part in the establishment of the present cottage hospital. We should like to see a spontaneous interest taken in the welfare of this institution by all classes of people. If this large industrial community feel that they would prefer to organise, as other places all over the country have organised, workmen's committees which will collect a definite sum per annum towards the cost of maintenance of this hospital they have it in their own power, if they wish, to render it unnecessary to charge each in-patient a definite sum.

In endeavouring to find a reason to account for the relatively low standard of manners and conduct of which complaint is made in regard to this community, on looking into the circumstances of the town and the extraordinary generosity exhibited towards it by men and women who have for the best part of a lifetime shown a noble liberality and public spirit on behalf of their fellows, our experience makes us wonder whether one cause may not be that in the past too much has been done for the people of Halstead, and too little insistence has been brought to bear upon the privilege of individual thrift, self-help, and independence. Be the cause what it may, it is time that those who lead opinion should set themselves conscientiously to arouse and bring about an active moral and spiritual awakening throughout the whole population. What better rallying point for this new departure could be found than a united, whole-hearted effort by all classes to cause the hospital better to subserve the wishes and circumstances of the people it serves so well. A few simple adjustments of its present system of admission should meet the case.

### THE CROMER COTTAGE HOSPITAL.

THIS hospital was established in 1866 and rebuilt in 1904. It is managed by a committee of fifteen ladies, and is so attractive, well-kept, and equipped as to make it one of the best of existing cottage hospitals. It was quite refreshing to go round this hospital and find everything of the best. It presents a marked and striking contrast to the structural defects of the Yarmouth hospital and to the mean and out-of-date equipment for the most part of the hospital at Lowestoft. It seems quite up to the standard maintained at the Norfolk and Norwich Hospital, and the committee of ladies and

A USEFUL TYPE—THE CROMER LOCKER.

the medical staff, especially Mr. Dent, who, with the late Mrs. Cooper and her daughter Miss Cooper, whose departure from Cromer is greatly regretted, have taken so leading a part in the history and efficiency of this institution for twenty years or more, deserve honour and thanks from the community which they have so ably and faithfully served. Recently Mr. George Bishop, R.N., has been appointed financial secretary, and we should hope he will speedily procure a copy of the matron's account books prepared on the uniform system, and take steps to keep the accounts in future on this system so that the expenditure and working of this excellent hospital may be comparable with those of other similar hospitals throughout the country.

The present matron, Miss Lambert, who first became matron twenty years ago and then went as matron to Guernsey Hospital, was engaged when we visited the hospital in the afternoon, and we did not therefore see her. We were taken round by one of the nurses who had been trained at Cromer, and who proved a most intelligent, knowledgeable, and interesting cicerone.

The hospital contains nineteen beds, with an average occupancy of eleven. The wards are most attractive in appearance and excellent from the hygienic point of view. The same may be said of the ward kitchens, lavatories, baths, beds, lockers, and entire equipment; indeed the lockers, which had glass tops, though made after a very simple pattern, from their proportions and design might well be adopted by the authorities of the Yarmouth, Lowestoft, and other hospitals. Through the courtesy of the matron we are able to give an illustration of the Cromer locker, which is well worth the consideration of hospital managers generally. It is simple, inexpensive, and efficient for its purpose. It is, we believe, 4 feet high by 1 foot 7½ inches broad. The cupboard proper is divided inside by a shelf, and there is an open space between the top of it and the top of the locker which is covered by ⅜-inch plate glass, for which it would be better to substitute encaustic tiles. In practice glass, however thick, is apt to wear badly as a covering for locker tops. At the back of the locker are a towel rail and a bracket. We have the details of this locker should any one be sufficiently interested to wish to have them.

The shelter and balconies attached to this hospital give evidence of the desire of the management to be well up to date and to see that their hospital should provide everything necessary to secure the best treatment and rapid recovery of the patients.

**The East Suffolk and Ipswich Hospital will be reported on next week.**

## Figures and Tables

**Figure f1:**
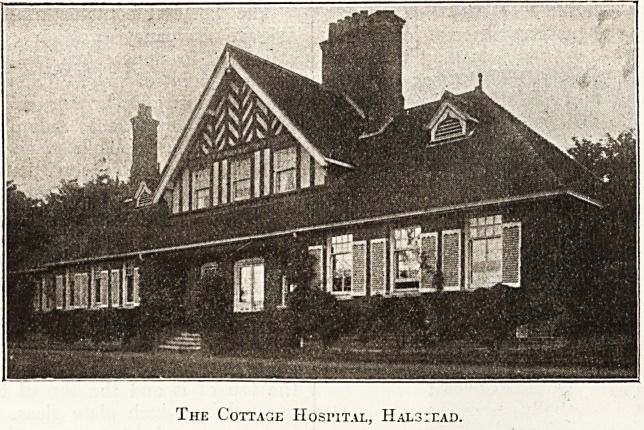


**Figure f2:**